# DC bias optimization in intelligent DCO-OFDM Li-Fi systems using hybrid machine learning with hardware validation

**DOI:** 10.1038/s41598-026-49732-4

**Published:** 2026-05-08

**Authors:** Esraa Abdelhakim, Dina A. Ragab, Mohamed Abaza, Mohamed Hussien Moharam

**Affiliations:** 1 Electronics and Communication Department, College of Engineering, Misr University for Science and Technology (MUST), P.O. Box 77, Giza, Egypt; 2 Electronics and Communication Engineering Department, Arab Academy for Science, Technology, and Maritime Transport, Giza, 12577 Egypt

**Keywords:** Li-Fi, DCO-OFDM, RMSE, R2-Score, MAPE, Energy science and technology, Engineering, Mathematics and computing

## Abstract

The main goal of this paper is to determine the optimal DC bias value for DC-biased optical orthogonal frequency division multiplexing (DCO-OFDM)- based Light fidelity (Li-Fi) systems using machine learning (ML) algorithms. The reason for this is that in DCO-OFDM, applying either a significant or a slight DC bias can lead to inefficiencies, such as increased clipping noise or reduced optical power. Therefore, it is crucial to determine the most appropriate DC bias level. So, to achieve this, ML algorithms were implemented. This paper compares ML techniques, including hybrid linear regression with K-Nearest Neighbors (KNN) and hybrid polynomial regression with KNN, for predicting the ideal DC bias value in DCO-OFDM-based Li-Fi systems. The models were evaluated based on their Root Mean Square Error (RMSE), the Coefficient of Determination (R2), and the Mean Absolute Percentage Error (MAPE). The results show that hybrid polynomial regression with KNN outperforms the other models, achieving a lower RMSE of 0.18847, a higher R2 of 96.908%, and a lower MAPE of 9.271%. In addition, to validate the model’s performance using real-world measurements, a hardware implementation of the Li-Fi system was developed using an Arduino-based receiver to collect actual signal data, which was then used to further evaluate prediction accuracy. The hardware results further confirmed the superiority of the hybrid polynomial regression with KNN model, achieving an RMSE of 0.296 and an R^2^ score of 81.37%, consistent with the simulation outcomes and demonstrating its robustness in real-time scenarios. These findings suggest that hybrid polynomial regression with KNN can be an effective technique for predicting the optimal DC bias value in DCO-OFDM-based Li-Fi systems.

## Introduction

Wireless fidelity (Wi-Fi) is now the most popular technology for delivering high-data-rate wireless Internet, and the need for high-speed wireless data is growing rapidly in the modern world. However, radio waves have drawbacks, including poor security and interference issues, and the frequency used by Wi-Fi is becoming more crowded, which raises the cost of spectrum licensing. To address these issues, Visible Light Communication (VLC) technology called Light Fidelity (Li-Fi) has been developed recently, which gives a data rate up to hundreds of gigabits per second^1^. Li-Fi is a wireless optical network technology that employs infrared light for uplink communication and visible light for downlink data delivery. It enables data communication alongside room illumination, providing a wireless networked scheme.

Li-Fi has significant advantages over Wi-Fi, including the use of large unregulated bandwidth in the optical spectrum, leading to data rates up to hundreds of gigabits per second, and is more secure and faster than Wi-Fi^2^. The data-carrying signal in Li-Fi does not pass through opaque walls, allowing it to be confined within a room, reducing interference and ensuring physical security. Furthermore, Li-Fi is more suitable for sensitive scenarios than Wi-Fi, especially in hospitals, as it does not have electromagnetic interference with medical equipment^3^. Compared with 5G, 6G wireless communication is projected to deliver improved spectral efficiency, privacy, energy efficiency, customization, and intelligence, while also supporting heterogeneous networks. Li-Fi may be a potential candidate for 6G since it provides secure high data rate communication in the license-free terahertz range^4^.

Li-Fi employs modulation methods that can be categorized into two distinct types: single-carrier and multi-carrier systems. Single-carrier systems use on–off keying (OOK), pulse-position modulation (PPM), and variable pulse-position modulation (VPPM); however, their primary drawback is reduced spectral efficiency, which reduces data transmission rates. Conversely, multi-carrier systems, including asymmetrically clipped optical OFDM (ACO-OFDM), DC-biased optical OFDM (DCO-OFDM), and hybrid diversity combined OFDM (HDC-OFDM), offer enhanced spectral efficiency^5^ DCO-OFDM is less power-efficient than ACO-OFDM in scenarios with constellations ranging from 4-QAM to 256-QAM, but it is more efficient in scenarios with larger constellation sizes, such as 1024-QAM and 4096-QAM^6^. However, the choice of DC bias in DCO-OFDM must be carefully selected to avoid high clipping noise and high optical power.

Existing DC bias selection methods are optimization-based; the use of a machine learning (ML) algorithm to identify an optimized DC bias value remains unexplored. ML algorithms are more computationally efficient and can find patterns, distributions, and trends in DCO-OFDM system data samples, giving them an edge over optimization approaches. ML algorithms applied to numeric datasets typically do not require high training time or computational hardware resources, which can be used to find the optimum DC bias value in DCO-OFDM-based Li-Fi^7^.

Although Li-Fi has gained considerable attention as a promising alternative to RF-based communication, optimizing the DC bias in DCO-OFDM Li-Fi systems remains a critical challenge. Several studies have applied conventional regression models to predict optimum bias values, often relying on MATLAB-generated datasets to evaluate performance. While these works provide valuable insights into the theoretical aspects of Li-Fi optimization, they do not fully capture the complexities of real-world deployment.

Despite these efforts, significant gaps remain. While^8^ demonstrated the effectiveness of machine learning algorithms for predicting the optimal DC bias in DCO-OFDM-based Li-Fi systems using simulation-generated datasets. Their analysis was limited to simulation-only validation. They neither explored hybrid regression models nor conducted real-world hardware testing, leaving open questions about actual performance under physical channel conditions.

The novelty of this work is threefold. First, we develop hybrid regression models that combine linear and polynomial regression with KNN, offering improved prediction accuracy compared to conventional standalone approaches. Second, we present an extensive comparative evaluation of multiple hybrid regression algorithms on MATLAB-generated datasets, demonstrating the superior accuracy of hybrid polynomial regression combined with KNN. Finally, unlike prior simulation-only studies, we implement a real-time Arduino-based Li-Fi hardware testbed and validate the proposed models with empirical measurements, proving the approach’s robustness under practical channel and noise conditions. This practical validation highlights the feasibility of deploying our proposed model in future Li-Fi networks, bridging the gap between academic research and real-world applications.

In this paper, various machine learning algorithms were used to determine the optimal DC bias value for DCO-OFDM in Li-Fi. We selected crucial features from the DCO-OFDM signal. We trained our model using various ML algorithms to identify the optimal DC bias value that yields the best performance for our proposed system. So, the main contributions of this work are the following:

- This work, according to the best of our knowledge, is the first to apply hybrid regression models to find the ideal DC bias value.
- Evaluating the effectiveness of using different ML hybrid regression algorithms to find the DC bias of DCO-OFDM on the MATLAB dataset.
- Implementing a real-time Li-Fi hardware setup to collect actual system data, which was used to validate the proposed machine learning models.

**Fig. 1:**
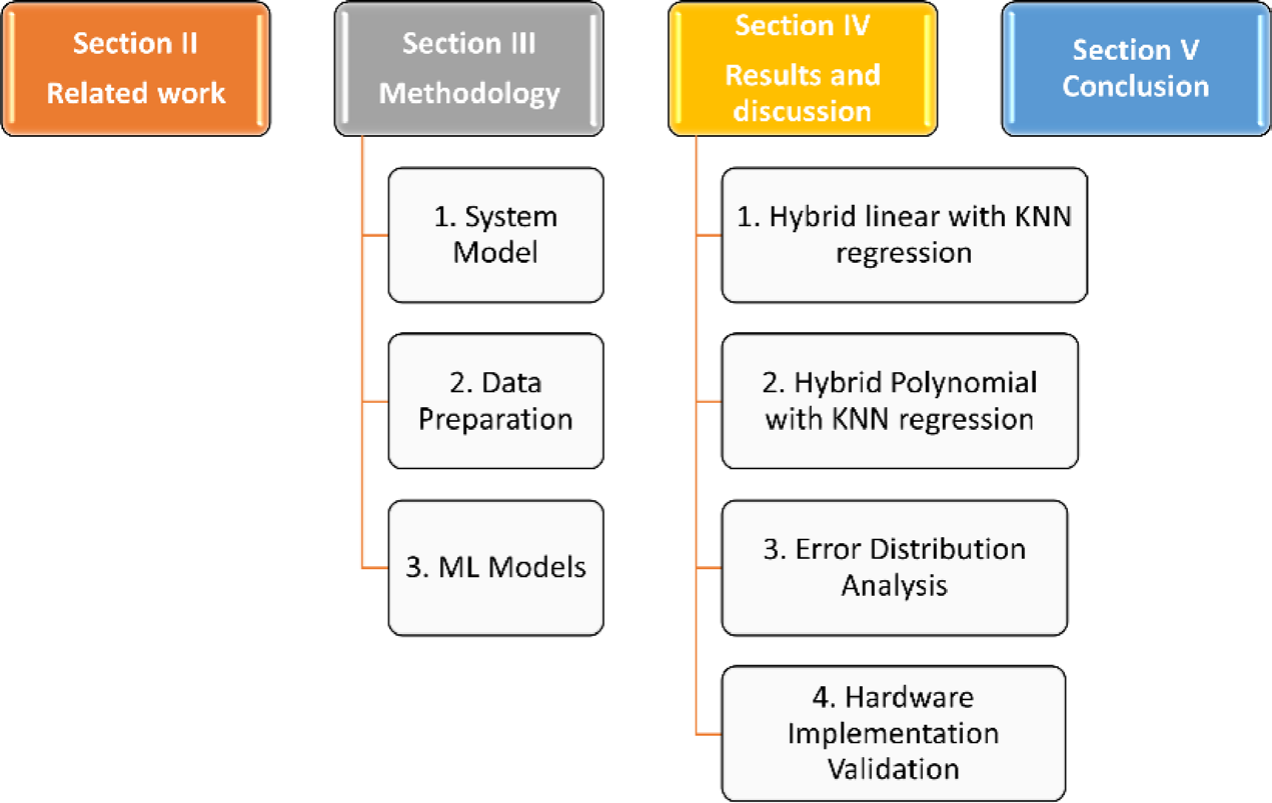
Paper structured.

The rest of the paper is structured as shown in Fig. 1. Related work discussion in Section II. The methodology, including the system model, data preparation, and ML models, is in Section III. Section IV presents the results and discussion for different hybrid regression models and the Hardware implementation validation. Finally, the conclusions in Sect. 5.

## Related work

Artificial intelligence (AI) refers to the ability of machines to perform cognitive functions similar to those of humans. Machine learning (ML) is a branch of AI where machines learn from datasets through specific programming. ML uses algorithms that improve as they process new data, building predictive models that can forecast outcomes for new data without changing the original programming. ML algorithms fall into two main categories: regression, which examines relationships between variables, and classification^9,10^, which categorizes data. ML is widely used in areas like data security, healthcare, finance, and transportation. In next-generation wireless communication, ML helps improve data transfer rates and is used for tasks like base station association, power management, spectrum management, network optimization, and more. Recent research shows that ML can enhance various aspects of telecommunications, including routing, localization, and spectral sensing, and reduce time.

There are numerous papers discussing DCO-OFDM and ML models. The study in^11^ examines an adaptive DC-biased Optical OFDM (DCO-OFDM) system as a complementary solution to acoustic communication for high-speed underwater data transfer. The proposed adaptive scheme is optimized to maximize system throughput while maintaining a desired target bit error rate (BER). At the receiver side, the signal-to-noise ratio (SNR) is determined for all subcarriers. Following this, all subcarriers can be assigned priority for data transfer, based on the maximum constellation size supported by all subcarriers and the target BER. Simulation results validate that the proposed link adaptation has remarkably improved system throughput, thereby proving its efficiency for high-speed underwater communication using adaptive DCO-OFDM.

Authors in^12^ Make a comparison between Hybrid Noise Cancelled Asymmetrically Clipped Optical OFDM (HNC-OFDM) and DCO-OFDM for optical wireless communication. The optimal DC bias, which is influenced by variables such as targeted BER and signal-to-noise ratio, plays a pivotal role in determining performance. Simulation results indicate that HNC-OFDM performs better in average-power-limited channels, whereas DCO-OFDM is more advantageous in peak-power-limited scenarios, underscoring its effectiveness and adaptability.

In^13^ The study highlights the role of DC-biased optical OFDM and asymmetrically clipped optical OFDM in enhancing Li-Fi technology. It focuses on these modulation techniques to improve bandwidth and performance in Li-Fi systems. It calls for further research to optimize them for practical use, aiming for greater energy efficiency and spectrum utilization. Authors at^14^ Underscore the significance of DC-biased optical OFDM for visible light communication (VLC) systems. The study highlights that DCO-OFDM, when paired with wavelength-division multiplexing (WDM) and adaptive methods, optimally uses bandwidth and reduces signal distortion, thereby improving the performance and feasibility of high-speed VLC applications.

The study in^15^ demonstrates that ML has numerous applications across many areas, and as such, it has been explored for optimizing optical communication systems. The relevance of ML is high when it is applicable for those tasks for which probabilistic techniques can deliver better performance compared to deterministic techniques, and also for those for which cost-effectiveness can be demonstrated compared to existing solutions. It is important to address various tasks in ML techniques, such as selecting the algorithm, making decisions during the training phase, monitoring, and adaptability during the implementation phase. Use cases of ML in the field of optical communication systems include: (1) Quality of Transmission (QoT) prediction in open and/or open/disaggregated systems, for which the performance can be obtained close to the vendor-specific implementation by integrating the low and high precision predictors and DNN calibration, and (2) Design of inverse Raman systems of high complexity, involving the determination of the pump power and wavelengths for realizing specific gain distributions, which can be solved by employing Autoencoders and GANs.

Authors in^16^ explore the benefits of optical networks for long-distance communication. It focuses on key performance metrics such as Quality factor, Optical Signal-to-Noise Ratio (OSNR), and Bit Error Rate (BER). The paper incorporates machine learning via an Adaptive Neural Fuzzy Inference System, designed to optimize network control by dynamically adjusting data rates based on path metrics such as OSNR.

The authors of^17^ proposed using machine learning (ML) to enhance the reliability and efficiency of VLC systems by exploring the wireless optical channel in indoor environments. The paper’s significant contribution is the efficiency achieved by using machine learning techniques to maximize VLC performance. This work underscores the potential of integrating ML into VLC to ensure stable, dependable communication in sensitive environments. In^18^ the paper explores the use of ML to enhance Li-Fi systems for indoor connectivity. By applying deep learning models such as multilayer perceptrons and convolutional neural networks (CNNs) to map the received signal-to-noise ratio, the study shows that these ML techniques significantly improve accuracy and efficiency compared to traditional methods, such as KNN.

The study in^19^ Presents a machine learning-based technique for synchronizing coherent optical OFDM systems. It employs a joint maximum-likelihood estimator to accurately estimate Timing Offset (TO), carrier frequency offset, and Carrier Phase Offset (CPO). This approach simplifies the estimation of TO and CPO and demonstrates superior performance compared to existing methods. In^20^ The authors suggest combining machine learning (ML) with Li-Fi technology to improve vehicle communication systems and reduce traffic accidents. This ML-driven model enhances safety by enabling more effective vehicle-to-vehicle and vehicle-to-infrastructure communication, aiming to prevent driver errors in restricted areas.

Authors in^21^ Presented a method for channel allocation in indoor VLC systems. It uses a probabilistic neural network (PNN) to classify VLC transmitters based on their proximity to the user’s position. The method allocates point-to-point channels after classification^22^, creating a secure communication zone that allows only authorized users to enter within a predetermined trust boundary. The algorithm’s ability to maintain this private area is validated by numerical simulations, which also demonstrate how ML improves VLC security by controlling user access and allocating channels effectively. In this study, different ML algorithms will be applied to optimize the DC bias value for DCO-OFDM Li-Fi systems.

The study in^23^ addresses one of the key challenges for VLC networks, namely, obstruction of links by moving objects, such as humans, which impedes the widespread use of indoor VLC networks. To address this challenge, the work proposes a predictive vertical handoff algorithm to enable seamless transition between Li-Fi and Wi-Fi networks. The algorithm uses a machine learning model to predict obstruction and actively manage network transitions, thereby optimizing the balance between Average Available Data Rate (AADR) and service loss. Through simulations that mimic link obstructions caused by human activity, the work demonstrates the algorithm’s efficacy in maintaining a high AADR while incurring minimal service loss. In addition, the algorithm’s adaptability enables flexible parameter configuration, allowing a range of service-reliability and data-rate trade-offs. These results validate the viability of combining predictive machine learning techniques in mixed-mode Li-Fi/Wi-Fi networks to improve indoor communication efficacy and offer a robust, flexible connectivity scheme.

To improve the security of visible light communications (VLCs), the authors in^24^ Investigate a transmission scheme that integrates computer-generated hologram (CGH)- based cryptographic coding with discrete Fourier transform (DFT)- precoded DCO-OFDM transmission. In the proposed scheme, information is encrypted using a double random phase approach and then encoded into a real-valued binary CGH using Lohmann coding. Then, DFT precoding is performed on a quadrature amplitude-modulated (QAM) symbol vector. Eventually, DCO-OFDM transmission is performed. Hence, the generated OFDM signal is then transmitted in VLC. In the simulation results, the authors found that the proposed secure transmission scheme provides high security and improves the PAPR of the generated OFDM signal and the system’s error performance in terms of BER. In addition, the proposed scheme is highly robust against noise and distortion. It provides a high correlation between the reconstructed and original images even at low SNR and with partial information loss.

While several recent studies have applied machine learning models such as SVR, GBR, and ensemble techniques to optimize DC bias levels in DCO-OFDM or related OFDM-based Li-Fi systems, many of these works rely solely on simulation datasets and do not extend their validation to real-time hardware environments. In addition, some methods focus primarily on improving accuracy metrics while overlooking the practical aspects of data generation and experimental feasibility. The present work addresses this gap by introducing a hybrid polynomial regression with a KNN approach, validated not only through MATLAB-based simulations but also through a custom hardware implementation of a Li-Fi link. This dual validation provides both theoretical robustness and real-world applicability. Table 1 summarizes the methodologies, datasets, and results reported in related works alongside our proposed model. It highlights that while prior approaches achieved competitive results in simulation, our model demonstrates superior accuracy and robustness in both simulated and real-time environments, thereby offering a more practical pathway for future Li-Fi deployment.

**Table 1:** Comparison between the proposed work and previous work.

Ref#	Methodology	Objective	Dataset used	Models & results	Limitation
^7^	Linear & polynomial regression (ML)	Predict optimum DC bias for DCO-OFDM Li-Fi	Simulation (MATLAB)	R^2^ score = 96.77%, RMSE = 0.19253, For Polynomial Regression R^2^ score = 0.8412%, RMSE = 0.4271, For Linear Regression	Simulation-only, no hybrid models, and no hardware validation
^25^	ML regressors (GBR, SVR)	Adaptive DC bias prediction	Simulation	GBR: R^2^ score = 97.9% SVR: R^2^ score = 92.2%	Only simulation, no hybrid or hardware validation
^26^	Machine learning–based DC-bias prediction + meta-heuristic optimization (XGBoost, DE)	Predict and optimize DC-bias to improve BER performance in ADO-OFDM systems	Simulation (MATLAB-generated dataset)	XGBoost achieved RMSE = 0.72 and R^2^ = 0.91 Differential evolution optimization improved BER by 58.62% (M = 64, SNR = 35 dB) compared to standard bias	Simulation-based study only; performance dependent on dataset quality and ML model generalization, with no experimental or hardware validation
^27^	Hybrid optical OFDM with ML regression (Polynomial regression)	Improve power efficiency and dimming flexibility in Li-Fi systems using a hybrid DPO-OFDM scheme	Simulation (MATLAB)	Polynomial regression of degree 4 achieved the best performance with R^2^ = 98.18% and RMSE = 1.18; DPO-OFDM achieved = 2 dB and 7 dB lower electrical power than HDAP and AHO, respectively, at BER = 10⁻^3^, with a dimming range of 8–92%	Focused on DPO-OFDM; simulation- Simulation-based evaluation only; ML models limited to regression for constellation size prediction, with no channel prediction or experimental validation
^28^	combination of Vandermonde-like matrix (VLM) precoding and nonlinear companding techniques (A-law, μ-law)	Reduce Peak Average Power Ratio (PAPR) in DCO-OFDM-based VLC systems	Simulation	PAPR reduction around 3.5 dB (by 76%) with acceptable BER and slightly higher SNR requirement	Simulation-only study, no AI/ML-based optimization, and no hardware validation
^29^	Multi-point constellation technique + discrete particle swarm optimization + selective mapping (MPC, DPSO, SLM)	Reduce PAPR in DCO-OFDM systems	Simulation	Proposed MPC combined with SLM reduced PAPR by up to 7 dB compared to conventional DCO-OFDM for 128 subcarriers and 16-QAM; outperformed conventional SLM for 4-QAM and 16-QAM	Simulation-based results only, with increased computational complexity due to constellation expansion and optimization, and no hardware validation
^30^	ML regressors (Random Forest, LazyPredict Algorithm for model selection)	Predict optimized DC bias for DCO-OFDM in Li-Fi with robust generalization	Simulation (MATLAB)	RF: R^2^ = 0.953, RMSE = 0.233. Model validated with Friedman test, hyperparameter tuning, bootstrap sampling for stability and robustness	Limited to simulation; dataset size could be increased for further robustness; no hardware validation
Proposed work	Hybrid regression (Linear regression with KNN, and Polynomial regression with KNN)	Predict optimum DC bias in DCO-OFDM Li-Fi	Simulation dataset (MATLAB) and Arduino hardware dataset	Simulation: R^2^ score = 96.908%, RMSE = 0.18847, For a hybrid Polynomial with KNN R^2^ score = 0.27513, RMSE = 96.427%, For a hybrid Linear with KNN) Hardware: R^2^ score = 81.37%, RMSE = 0.296, For a hybrid Polynomial with KNN) R^2^ score = 79.16%, RMSE = 0.313, For a hybrid Linear with KNN	Demonstrated real-time hardware viability and robustness through practical validation

## Methodology

### System model

**Fig. 2:**
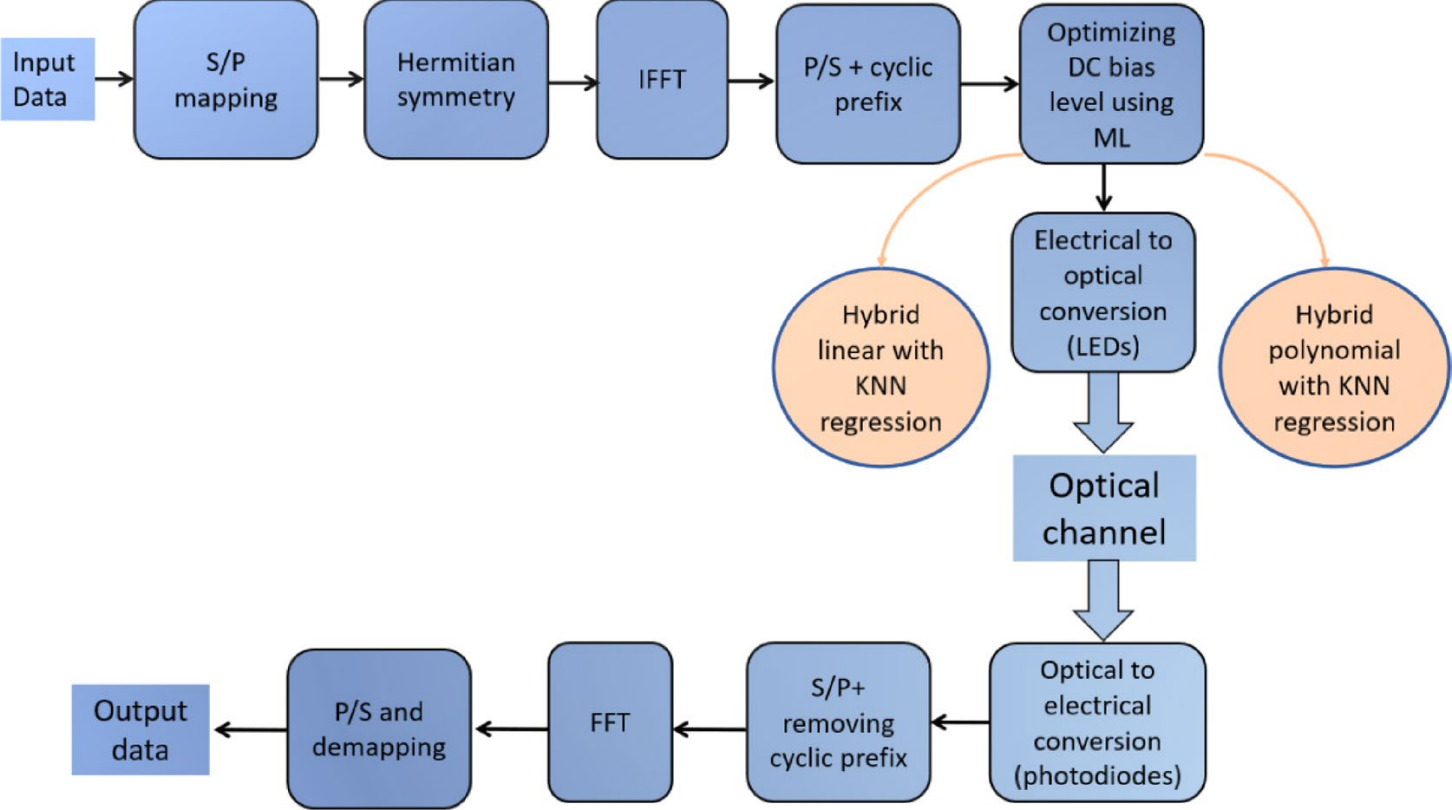
The proposed system model for optimizing DC bias level in DCO-OFDM for Li-Fi systems.

The proposed system model for optimizing DC bias level in DCO-OFDM for Li-Fi systems using different ML algorithms is shown in Fig. 2. The input data in the DCO-OFDM system is mapped into a constellation of complex values following encoding. The inverse fast Fourier transform (IFFT) is then used to convert the complex data symbols into a time-domain signal. However, Hermitian symmetry in the complex data is required to guarantee that the output of the IFFT is real. This symmetry guarantees that, for $0<K<N/2$, S_*K*_ is equal to the complex conjugate of S_*N-K*_*, and* the two components S_0_ and S_*N/2*_ are set to zero. After the OFDM signal is generated, a cyclic prefix is added to protect it from inter-symbol interference. But because OFDM signals have a high peak-to-average power ratio, certain peaks can be much higher than the average. A significant DC bias is needed to eliminate negative peaks, but the cost in optical power is a crucial consideration. Thus, for DCO-OFDM, an ideal DC bias is required, which can be optimized using various ML techniques. So, hybrid linear with KNN regression and hybrid polynomial with KNN regression were used on the modulated signal.

Next, the modulated signal is converted from an electrical to an optical signal. The optical signal is transmitted via the optical channel, then detected, and the electrical signal is restored at the receiving end. The original data is then demodulated using the FFT technique after the signal has been filtered to eliminate any unwanted noise.

### Data preparation

**Fig. 3:**
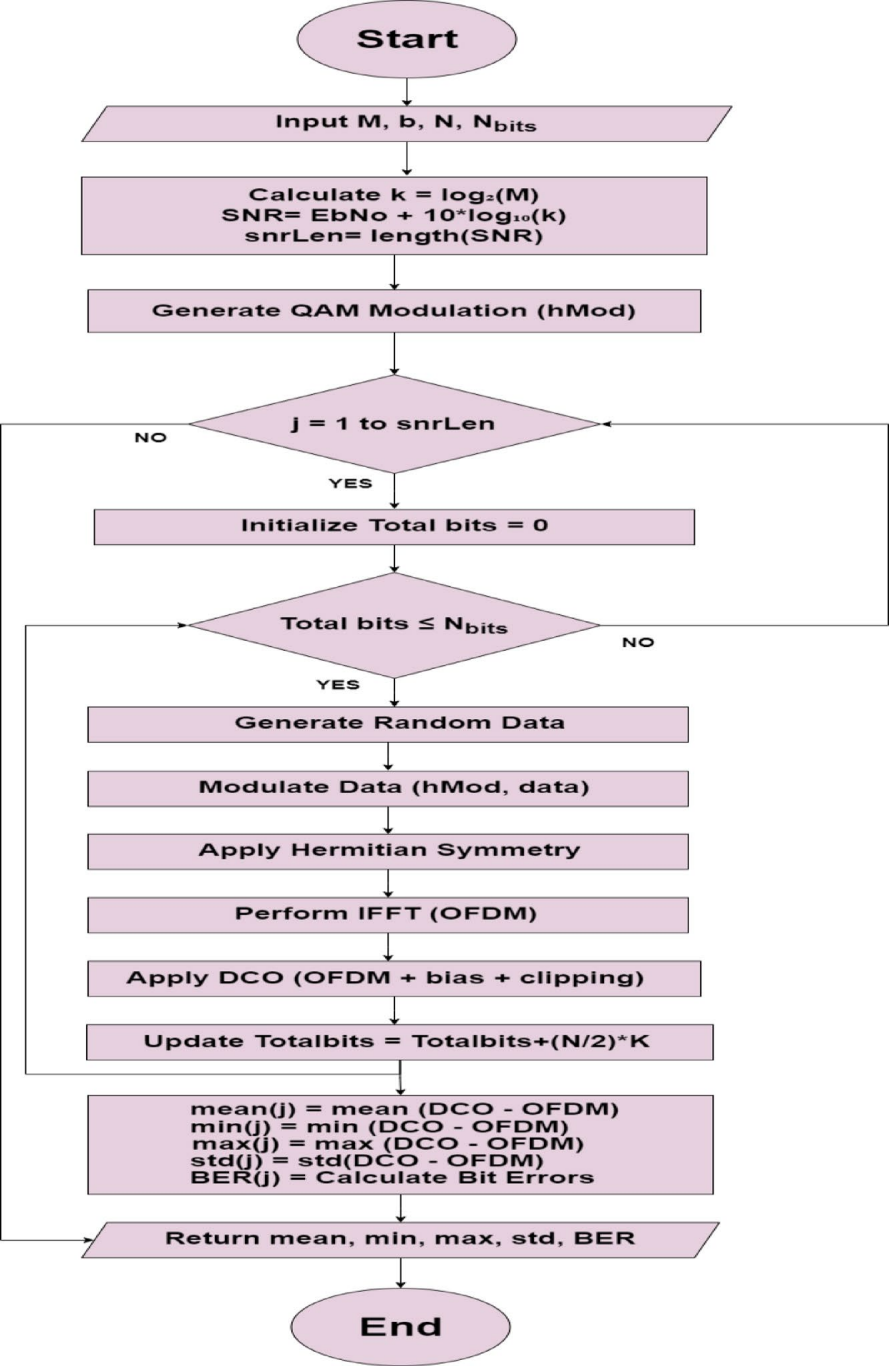
Flowchart of data preparation for DCO-OFDM systems from MATLAB.

To find the ideal DC bias using ML algorithms, a dataset was generated for DCO-OFDM systems using MATLAB version 9.13.0 (R2022b)^31^ In an HP laptop with a 2.50 GHz, 2.70 GHz dual-core Intel Core i5 processor and 8 GB RAM. The generation of DCO-OFDM signals relies on several parameters, such as the constellation size (M), which represents the number of bits per symbol, and the energy per bit to noise spectral density (Eb/N0). Initially, the data was mapped to quadrature amplitude modulation (QAM) constellation points while maintaining Hermitian symmetry for the complex values. Afterward, an inverse fast Fourier transform (IFFT) was applied, and due to the Hermitian symmetry, the IFFT output was a real-valued signal. This output was a bipolar OFDM signal, which was then biased by adding a DC component, with any remaining negative values clipped to produce a unipolar DCO-OFDM signal. A variety of OFDM signals were generated with different parameters. From these signals, key statistical features, including the mean, minimum, maximum, standard deviation, and bit error rate (BER), were calculated to form the dataset. The MATLAB flowchart for data generation for DCO-OFDM systems is shown in Fig. 3.

Where M stands for signal constellation size, b for bias values, N for subcarriers, Nbits for the number of bits to be processed, and min: the OFDM signal’s minimum value, max: OFDM signal maximum value, std: OFDM signal standard deviation, mean: mean value of OFDM signal, and BER: bit error rate.

### ML models

A MATLAB simulation of the DCO-OFDM system was used to extract various parameters, such as the mean, min, max, std, BER, M, bias, and N, to create a dataset, as shown in Table 2. The dataset consists of 250 samples, and eight parameters were produced by varying the DC-biased value, constellation size, and number of sub-carriers. The dataset’s records were randomly shuffled for use with Python to apply ML models.

**Table 2:** Dataset parameters.

Parameter name	M	Mean	Min	Max	Std	BER	Bias	N
Serial	1	2	3	4	5	6	7	8

**Fig. 4:**
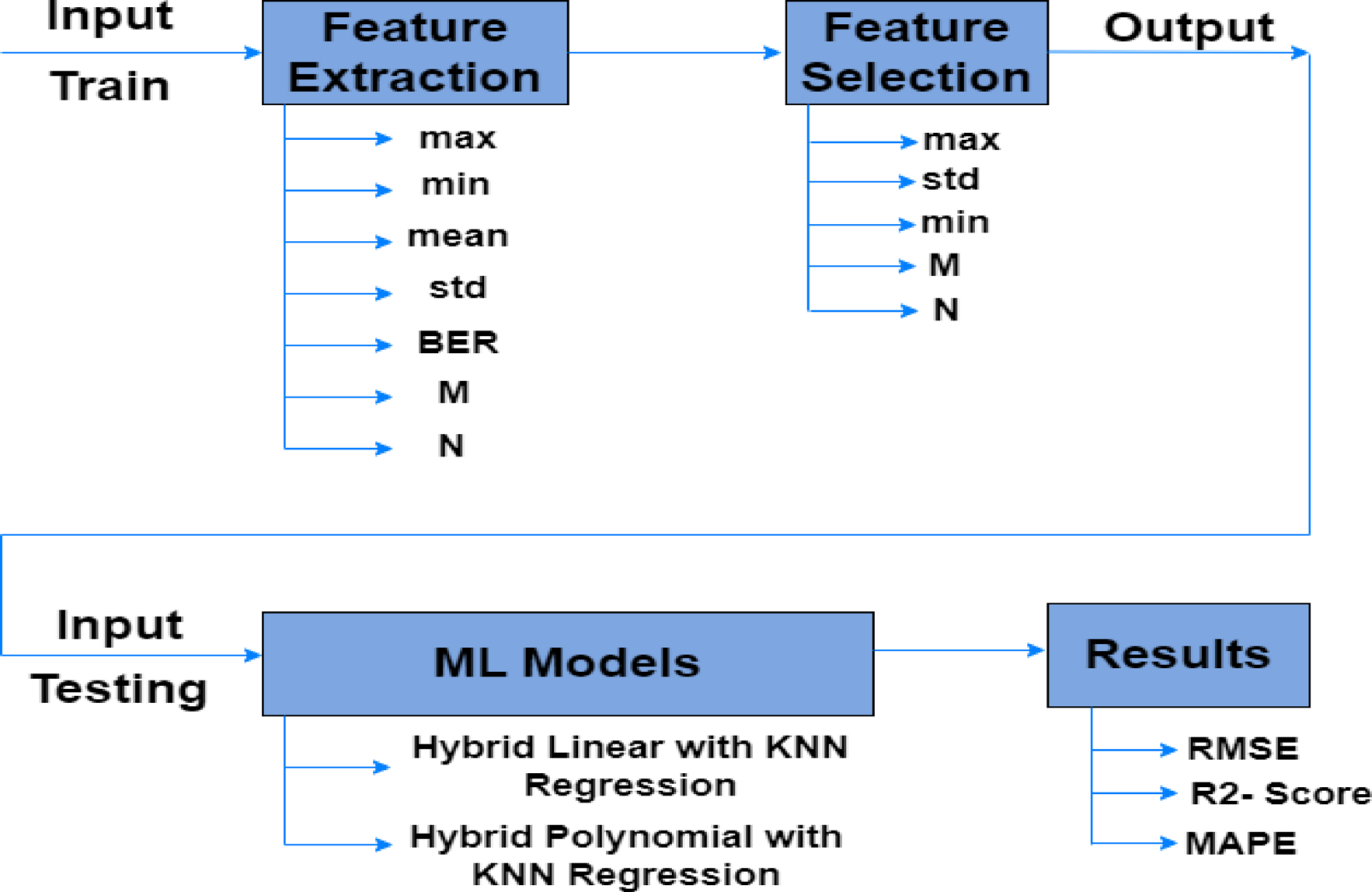
Steps for applying ML models to the MATLAB dataset.

The dataset was used to train multiple machine learning algorithms to maximize accuracy and identify the optimal DC bias value. With a splitting data by 70%:30% which is the most common ratio used in ML models and gives the best performance^32^. This separation made it possible to train the model efficiently, ensuring it captured patterns in the data, and to test it on a different sample to measure its effectiveness. The testing dataset offers an objective assessment of the model’s accuracy, while the training dataset was used to optimize the model’s parameters. After data splitting, Scikit-Learn feature selection techniques were used to find the most appropriate features for predicting the DC bias value. Specifically, the SelectKBest method with F-regression scoring is used to select the most critical features for the OFDM signal. The five most important features were M, min, max, std, and N, as shown in Table 3. Then, two hybrid ML techniques, hybrid linear with KNN regression and hybrid polynomial with KNN regression, were applied to find the best result. Figure 4 shows the steps to be followed when applying ML models to optimize DC bias.

**Table 3:** Parameters with scoring.

Parameters	Scoring
Max	461.408
Std	427.578
Min	300.399
M	241.632
N	101.188
BER	6.118
Mean	1.1421

Different hybrid ML models were applied and want to know which model is the best among them. So, the performance was evaluated by measuring root mean square error (RMSE) as in Eq. (1)^33^, coefficient of determination (R2 score) given by Eq. (2)^34,35^, and mean absolute percentage error (MAPE) as in Eq. (3)^36^.

1 $$RMSE = \sqrt {\frac{1}{n}\sum\limits_{i = 1}^{n} {(y_{i} - \hat{y}_{i} )^{2} } }$$

2 $$R^{2} = 1 - \frac{{\sum\nolimits_{i = 1}^{n} {(y_{i} - \hat{y}_{i} )^{2} } }}{{\sum\nolimits_{i = 1}^{n} {(y_{i} - \overline{y}_{i} )^{2} } }}$$

3 $$MAPE = \frac{1}{n}\sum\limits_{i = 1}^{n} {\left| {\frac{{y_{i} - \hat{y}_{i} }}{{y_{i} }}} \right|} \times 100$$

where $n$ The total number of predictions, ${y}_{i}$ the actual value, ${\widehat{y}}_{i}$ the predicted value, and ${\overline{\mathrm{y}} }_{i}$ The mean (average) of the actual values.

## Results and discussions

### Hybrid linear with KNN regression

Initially, the number of neighbors (n-neighbors) for KNN regression was set to 1. The number of features (k) varied from 1 to 7. For each configuration, the RMSE, R2 score, and MAPE were calculated to evaluate model performance. Subsequently, the number of neighbors (*n*-neighbors) increased to 3, and the number of features was adjusted again. The same evaluation metrics, RMSE, R2 score, and MAPE, were computed for this new configuration to ensure consistency in the performance assessment. Finally, the number of neighbors (*n*-neighbors) was set to 5, and the process of varying the number of features and evaluating the same metrics was repeated. The objective was to determine the optimal configuration that yields the highest R2 score and the lowest RMSE and MAPE values.

**Algorithm 1:**
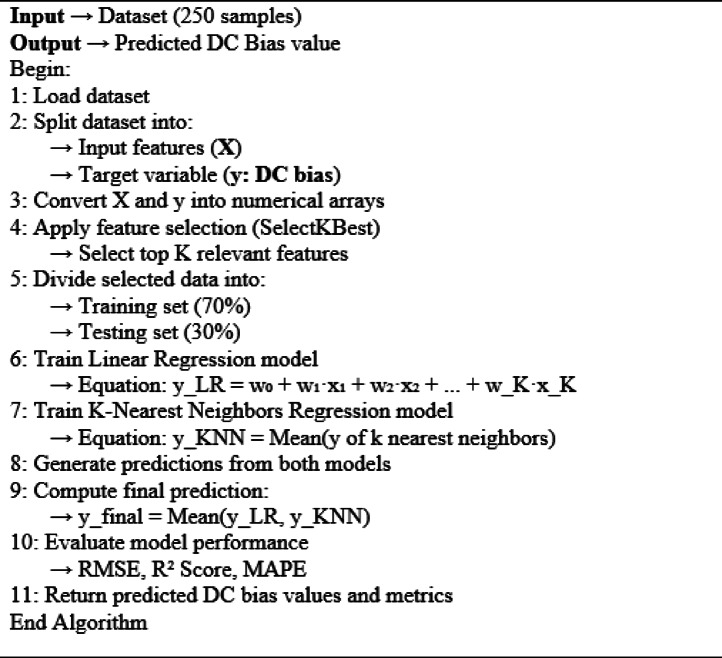
Hybrid linear regression with KNN model for DC bias prediction.

Table 4 shows the three measured metrics (R2 score, RMSE, and MAPE) for each configuration, varying the number of neighbors (*n*-neighbors) and the number of features. As shown in Table 4, increasing the number of features generally improves the performance of the KNN regression model. This improvement was evident across all values of n-neighbors. For example, with n-neighbors set to 1, the RMSE decreased from 0.3267 with 1 feature to 0.2026 with 5 features. Similarly, the R2-Score improved from 0.9071 to 0.9643, and the MAPE decreased from 18.0061 to 9.7031. However, after reaching five features, additional features provide diminishing returns in performance improvement.

**Table 4:** RMSE, R2-score, and MAPE for the hybrid linear with KNN regression on the MATLAB dataset.

n-neighbors	Features, K	RMSE	R2-Score	MAPE
1	1	0.32666	0.90715	18.00613
	2	0.32473	0.90824	17.83617
	3	0.32277	0.90935	17.70387
	4	0.31937	0.91124	17.11834
	5	0.20263	0.96427	9.70312
	6	0.20433	0.96367	9.31413
	7	0.20438	0.96365	9.34815
3	1	0.40369	0.85820	20.71674
	2	0.39237	0.86603	18.77828
	3	0.38229	0.87283	18.89255
	4	0.34866	0.89422	17.62278
	5	0.24623	0.94724	11.72208
	6	0.24674	0.94702	11.43298
	7	0.24676	0.94702	11.43958
5	1	0.42773	0.84080	21.09920
	2	0.42445	0.84323	19.69907
	3	0.40828	0.85495	18.83617
	4	0.35010	0.89334	17.35041
	5	0.25368	0.94400	11.89399
	6	0.25496	0.94343	11.65602
	7	0.25497	0.94343	11.66520

Also, the results indicate that the number of neighbors significantly impacts the performance metrics. When comparing different values of n-neighbors for the same number of features, n-neighbors equal to 1 often yield the best performance. For instance, with five features, the RMSE is 0.2026 for n-neighbors equal to 1, compared to 0.2462 and 0.2537 for n-neighbors equal to 3 and 5, respectively. Similarly, the R2-Score and MAPE show that n-neighbors equal to 1 generally yield higher accuracy and lower errors.

The experimental results demonstrate that for optimal performance of the KNN regression model, using five features with n-neighbors equal to 1 provides the best balance of accuracy and error minimization, resulting in the lowest RMSE of 0.20263 and the highest R2-Score of 0.96427. Increasing the number of features beyond 5 yields marginal improvements and using more than 1 neighbor generally results in higher RMSE and MAPE values, and lower R2-Scores.

**Fig. 5:**
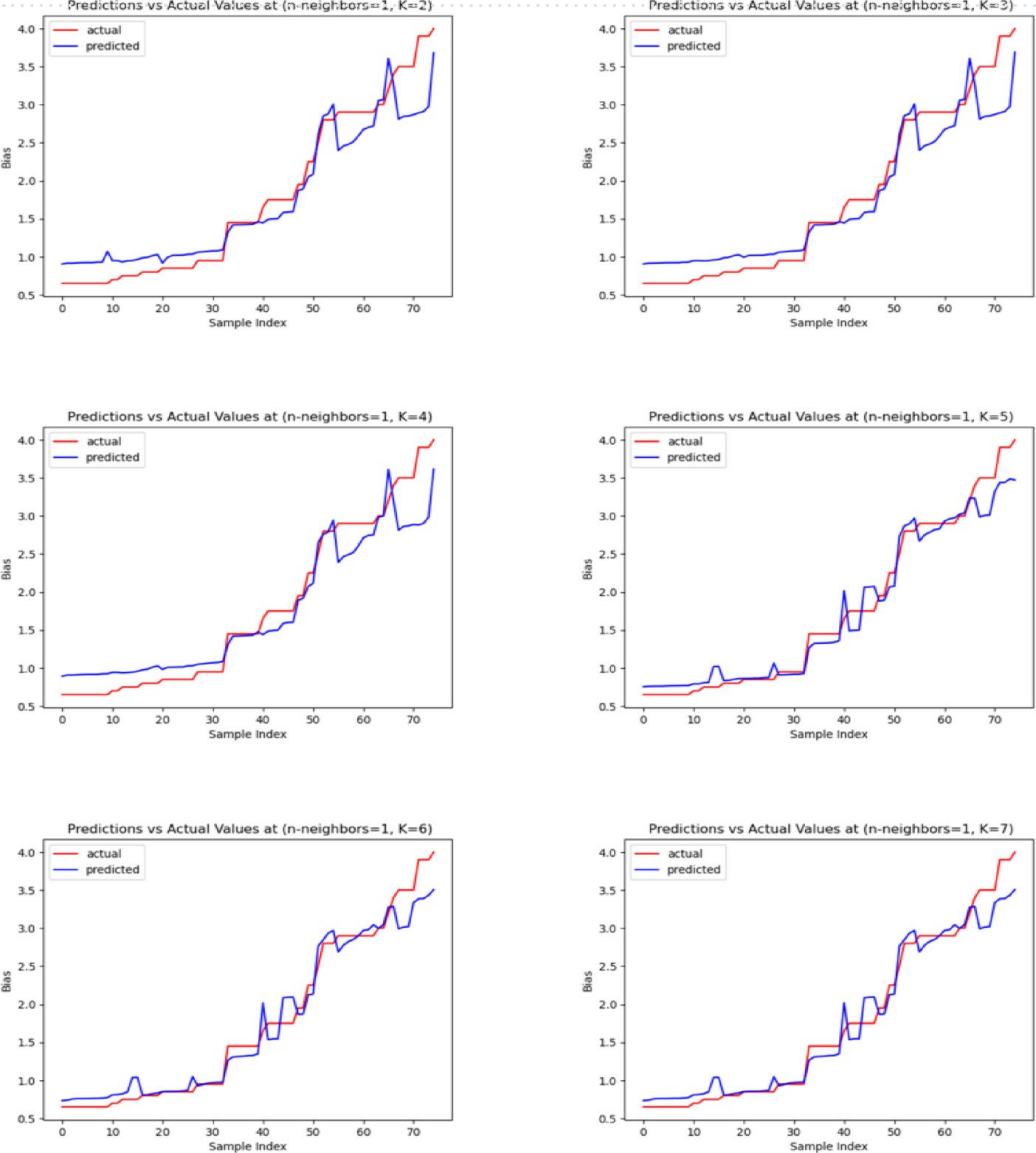
Actual and predicted bias values for the hybrid linear with KNN regression from the MATLAB dataset.

In Fig. 5, the predicted and actual DC bias values are presented for a hybrid linear model with a KNN regression with n-neighbors equal to 1 and varying numbers of features to demonstrate the effectiveness of the hybrid model. From Fig. 5 we found that the actual value of the DC bias and the predicted value of the DC bias are well-aligned, especially at k equal to 5. This close alignment indicated that the hybrid linear-KNN regression model was effective in accurately predicting the appropriate DC bias value for DCO-OFDM-based Li-Fi systems.

### Hybrid polynomial with KNN regression

For the case of a hybrid polynomial with KNN regression, we compute the same metrics: R2 score, RMSE, and MAPE, varying the polynomial degree (p), the number of neighbors (*n*-neighbors), and the number of features (K).

**Algorithm 2:**
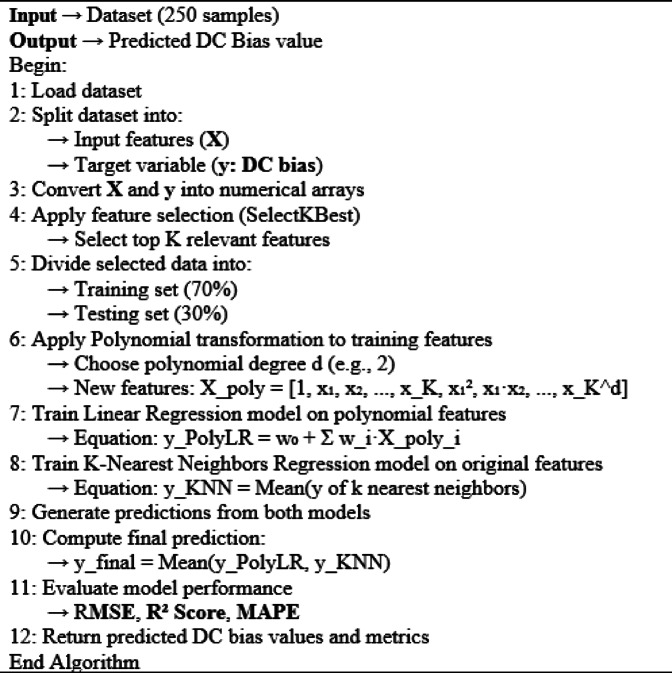
Hybrid polynomial regression with KNN model for DC bias prediction.

Table 5 shows the results for the three metrics as the polynomial degree (p) varies from 1 to 3, with n-neighbors equal to 1, 3, and 5, and features ranging from 5 to 7. From the results shown in Table 5, the optimal performance of the hybrid polynomial with the KNN regression model by using seven features with n-neighbors equal to 5 and degree of 2, which provided the best balance of accuracy and error minimization, with the lowest RMSE of 0.18847 and the highest R2-Score of 0.96908.

**Table 5:** RMSE, R2 score, and MAPE for the hybrid polynomial with KNN regression on the MATLAB dataset.

Degree of polynomial, p	n-neighbors	Features, K	RMSE	R2-Score	MAPE
1	3	5	0.27386	0.93474	13.00724
		6	0.27537	0.93401	12.73673
		7	0.27538	0.93401	12.75126
	5	5	0.28031	0.93163	13.00376
		6	0.28182	0.93089	12.71461
		7	0.28184	0.93088	12.72499
2	3	5	0.20156	0.96465	9.78572
		6	0.18928	0.96882	9.37518
		7	0.18923	0.96884	9.37372
	5	5	0.20440	0.96364	9.74439
		6	0.18851	0.96908	9.27520
		7	0.18848	0.96909	9.27178
3	3	5	0.18882	0.96898	9.40420
		6	0.23770	0.95084	9.99542
		7	0.26983	0.93665	10.98321
	5	5	0.19051	0.96842	9.25554
		6	0.23260	0.95292	10.01421
		7	0.26778	0.93761	11.00103

**Fig. 6:**
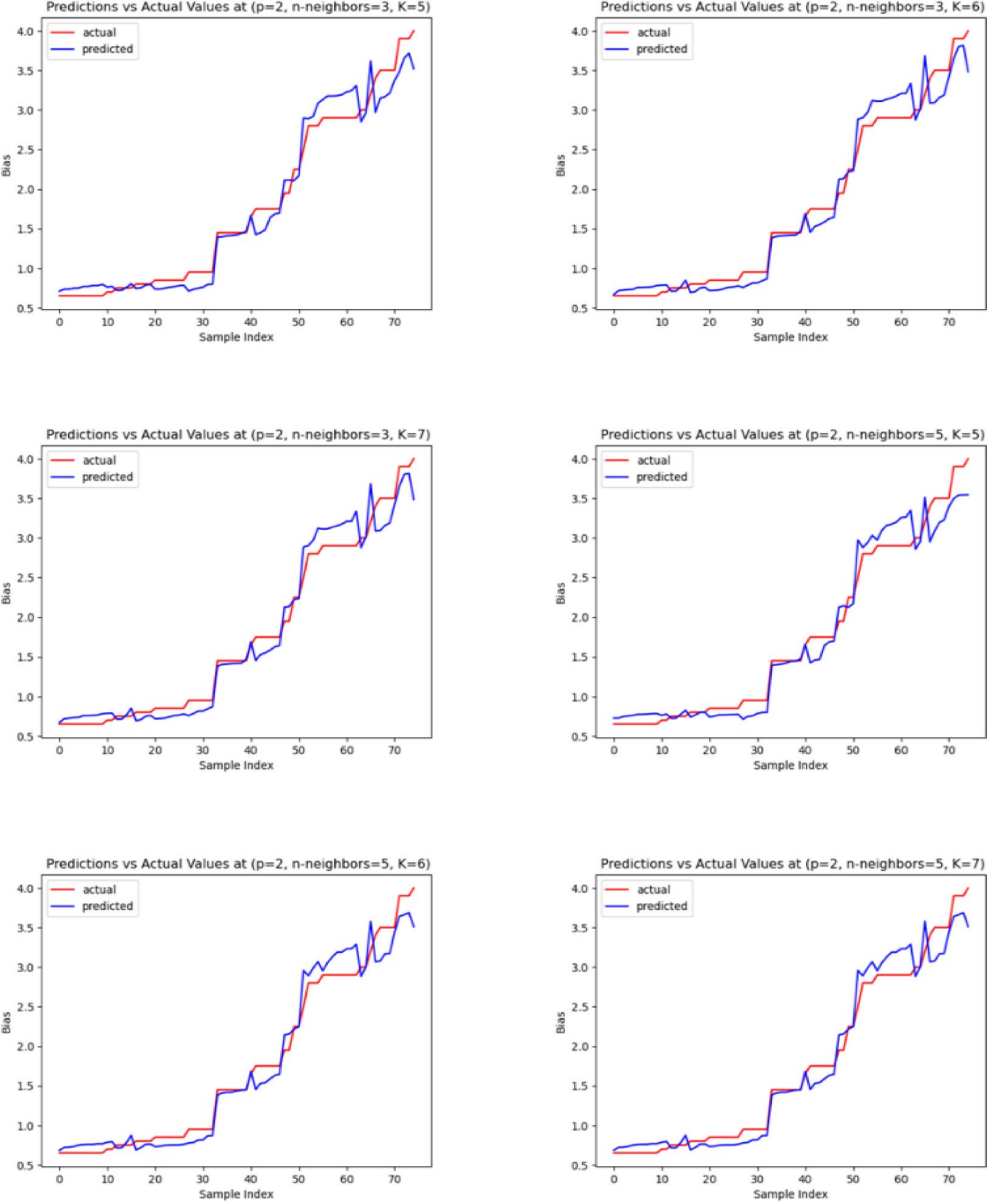
Actual and predicted bias values for the hybrid Polynomial with KNN regression from the MATLAB dataset.

The predicted and actual DC bias values for the hybrid polynomial with the KNN regression model are shown graphically in Fig. 6. From Fig. 6, the predicted value is too close to the actual DC bias value, indicating that the model successfully selected the DC bias value using seven features with n-neighbors equal to 5 and degree 2.

### Error distribution analysis

**Fig. 7:**
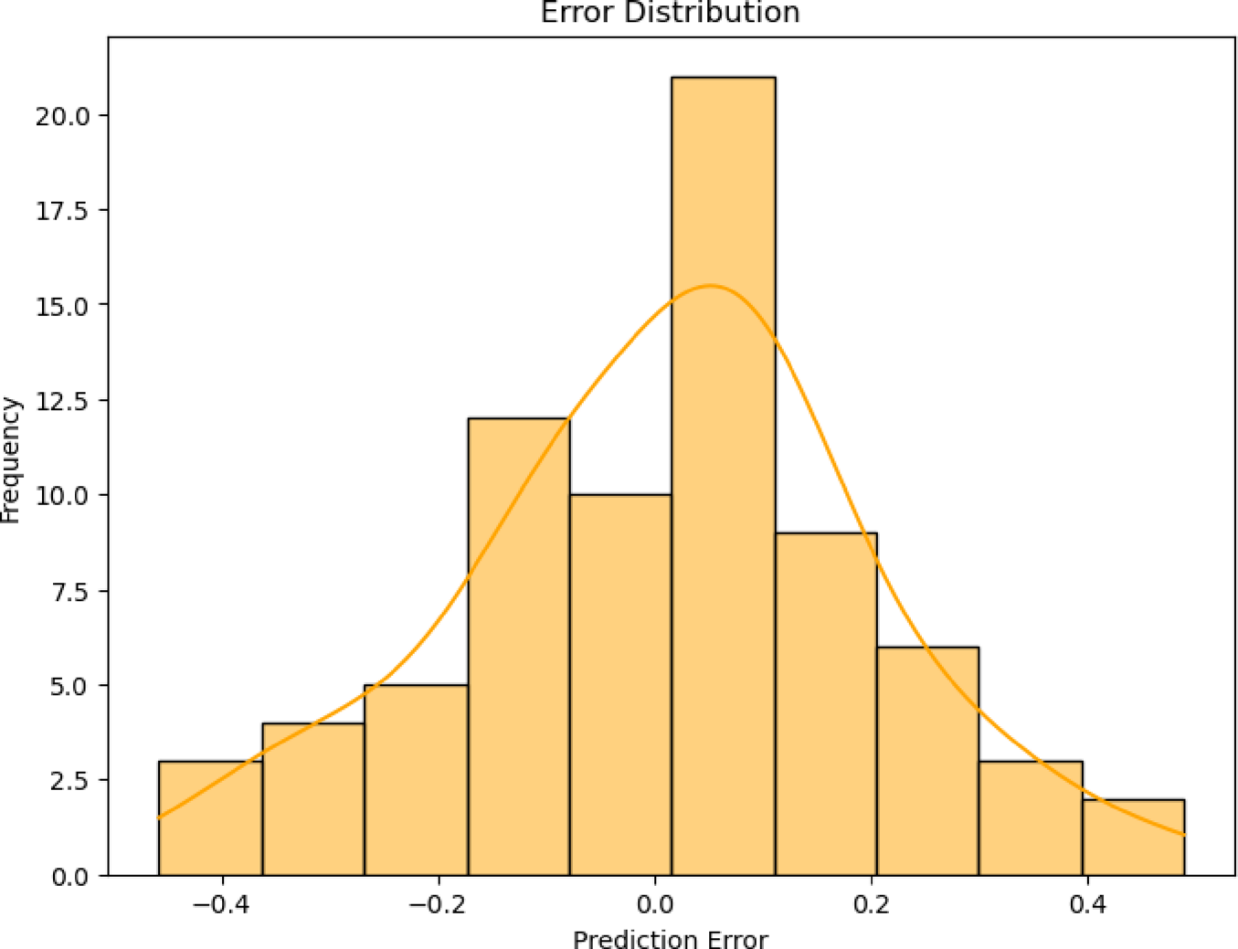
Error distribution for a hybrid polynomial with KNN regression.

The spread of prediction errors produced by the hybrid polynomial with KNN regression was illustrated in Fig. 7. This storyline offers a perspective on the differences between the model’s real and projected values. The prediction error is shown on the x-axis, with values from around -0.4 to 0.4, and the frequency of these errors is shown on the y-axis. Most prediction errors cluster near zero, indicating that the hybrid model accurately predicts in most cases.

The Kernel Density Estimation (KDE) curve overlaid on the histogram also lends further support, offering a smooth estimate of the probability density of the error distribution. The peak of the KDE curve is close to zero, indicating strong model performance with minimal errors. Additionally, the error distribution appears fairly balanced, suggesting that the model does not exhibit a consistent tendency to overestimate or underestimate. The even distribution of mistakes indicates how the hybrid method successfully merges the advantages of the hybrid Polynomial with KNN Regression, resulting in enhanced predictive accuracy. In general, the storyline shows that the hybrid model’s forecasts are correct, with most errors minor and evenly distributed around zero.

**Fig. 8:**
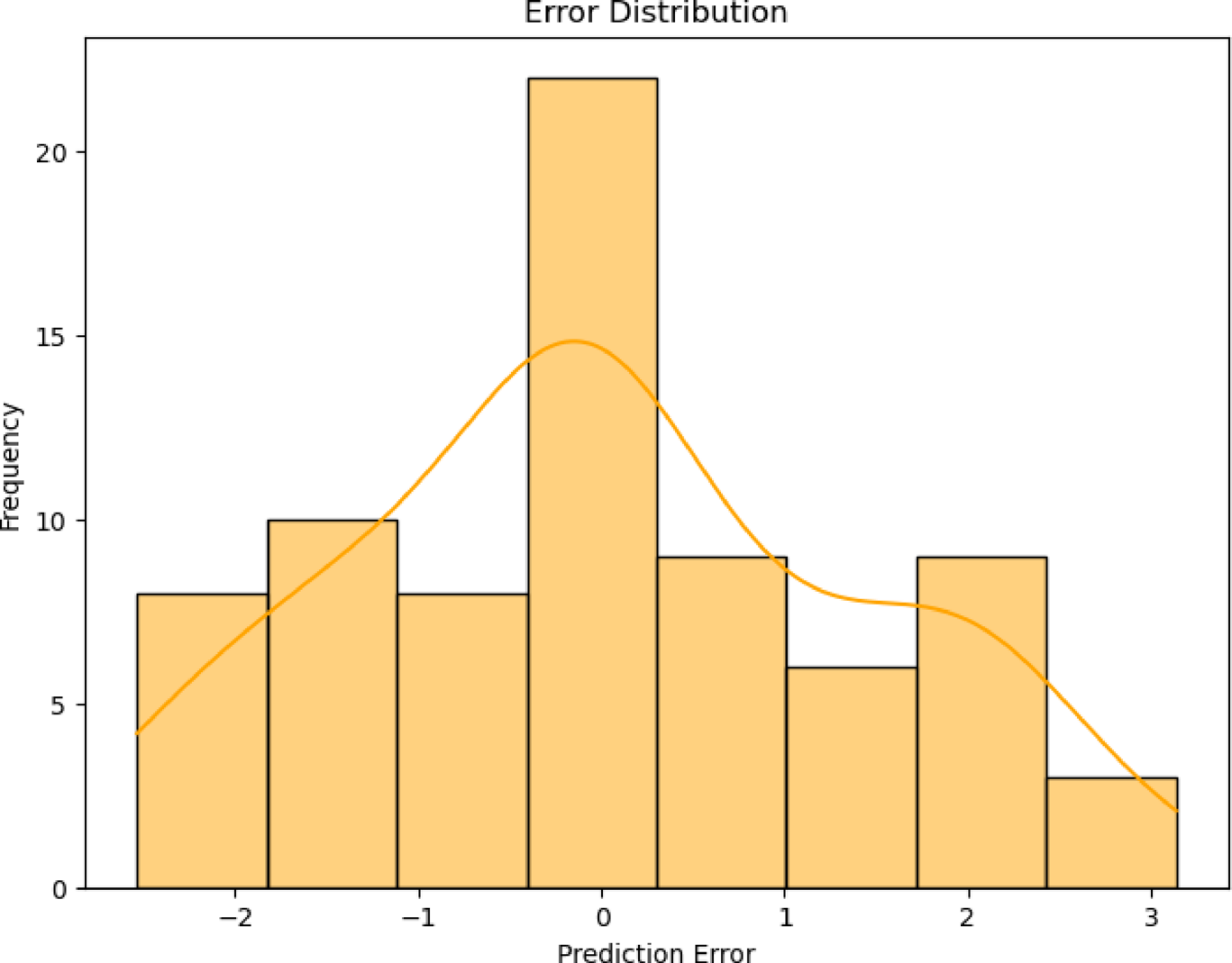
Error distribution for hybrid linear with KNN regression.

The error distribution of the hybrid Linear Regression with KNN Regression was presented in Fig. 8. Unlike the more tightly clustered error distribution seen in Fig. 7, the error range in this model is significantly wider, extending from -2 to 3. This broader spread suggests that the system produces more substantial errors, especially in the positive direction, where the model appears to overestimate.

The KDE curve in this case shows multiple peaks, indicating potential variations in model performance under different conditions. Overall, the model’s performance seems less consistent than that of the hybrid Polynomial with KNN Regression approach. So, the first model demonstrates better accuracy with most errors clustering near zero, while the second model shows a broader range of errors, suggesting less precision.

### Hardware implementation validation

To enhance the reliability and practical value of the proposed models, this section documents a newly added validation phase based on real-time hardware measurements. The motivation for this addition was to verify the accuracy of the machine learning models in real-world scenarios, compare their performance against data collected from a hardware implementation of a Li-Fi system, and assess their robustness beyond simulation environments.

**Fig. 9:**
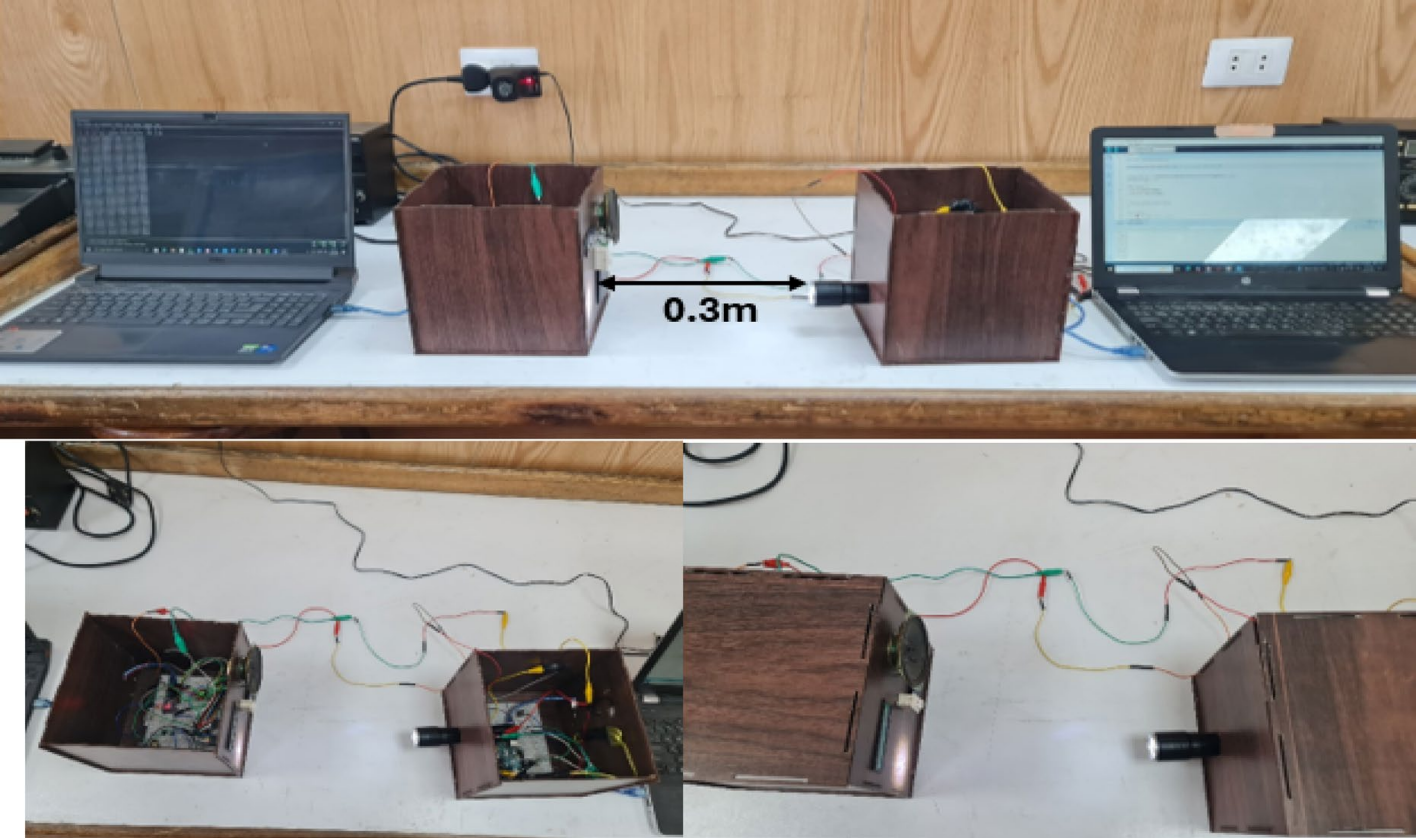
Real-time hardware setup for DCO-OFDM-based Li-Fi system.

To implement the real-time Li-Fi transmission system, a hardware setup was constructed using a high-brightness LED as the optical transmitter and a photodiode as the optical receiver at a distance of 0.3 m, as shown in Fig. 9. The LED was modulated with DCO-OFDM signals, while the photodiode captured the received optical signal and converted it into an electrical signal. The output was processed by an analog front-end circuit connected to an Arduino microcontroller, which sampled and recorded the signal data.

The Arduino collected the received signal parameters and exported them to a CSV file via the serial interface. This data was then analyzed and used as input to the same machine learning models used on the MATLAB dataset, with the same dataset size of 250 samples and eight parameters. The setup was designed to closely emulate the conditions of a Li-Fi communication system under practical constraints.

To evaluate the effectiveness of the proposed hybrid ML models under real-world conditions, the same trained models from the simulation phase were applied directly to the hardware-generated dataset. The hardware data was formatted to match the simulation feature structure, including parameters such as mean, minimum, maximum, standard deviation, bit error rate, and the applied DC bias. This ensured that the models could process the new data without any retraining or reconfiguration.

The results showed that the hybrid polynomial regression with KNN continued to outperform the hybrid linear regression with KNN on the hardware data. It provided more accurate predictions, with lower errors and better correlation to the actual DC bias values. This demonstrates that the hybrid polynomial with the KNN model is not only effective in simulation but also robust and reliable in real-world scenarios.

**Fig. 10:**
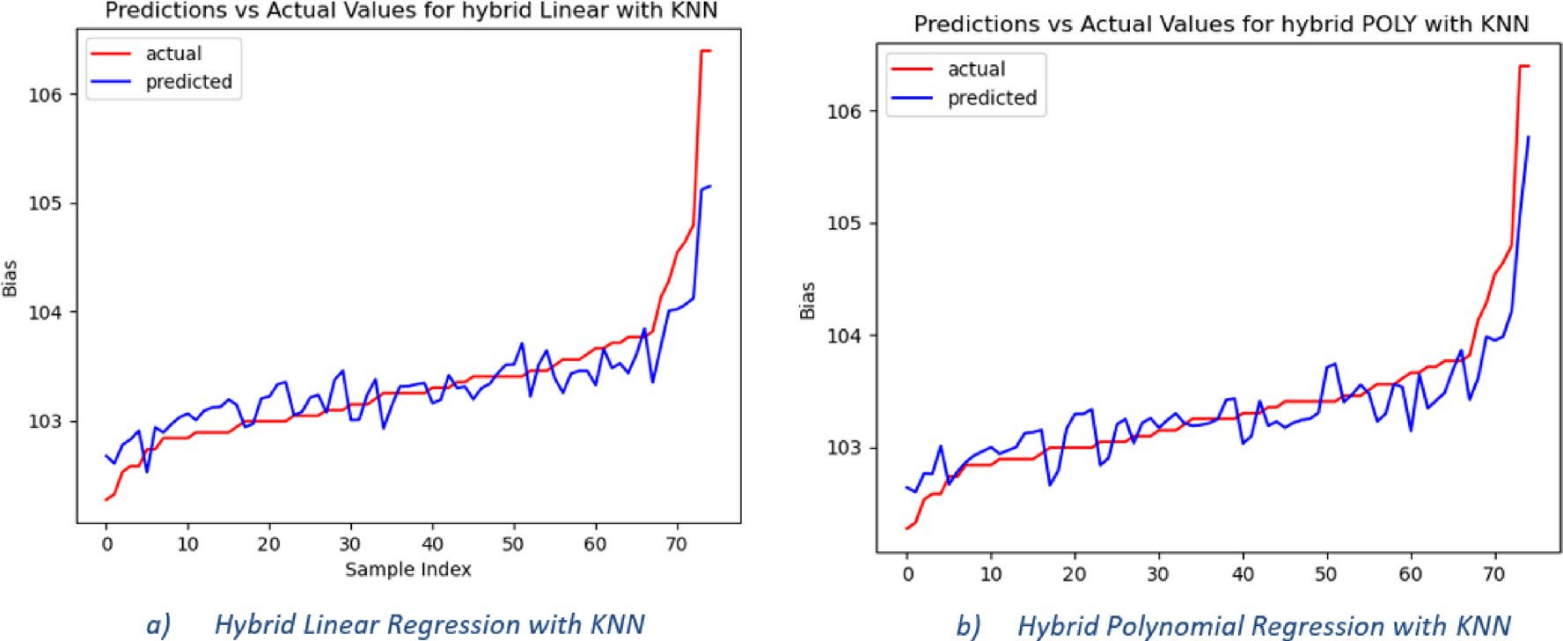
The actual and predicted bias values for the two hybrid models of real-time hardware data.

These findings confirm the generalizability of the proposed approach, validating that the trained models can accurately predict the optimal DC bias in DCO-OFDM-based Li-Fi systems using both simulated and real-time hardware data. Figure 10 shows the actual and predicted bias values for the two hybrid models’ real-time hardware data: a) hybrid linear with KNN, b) hybrid Polynomial with KNN.

**Fig. 11:**
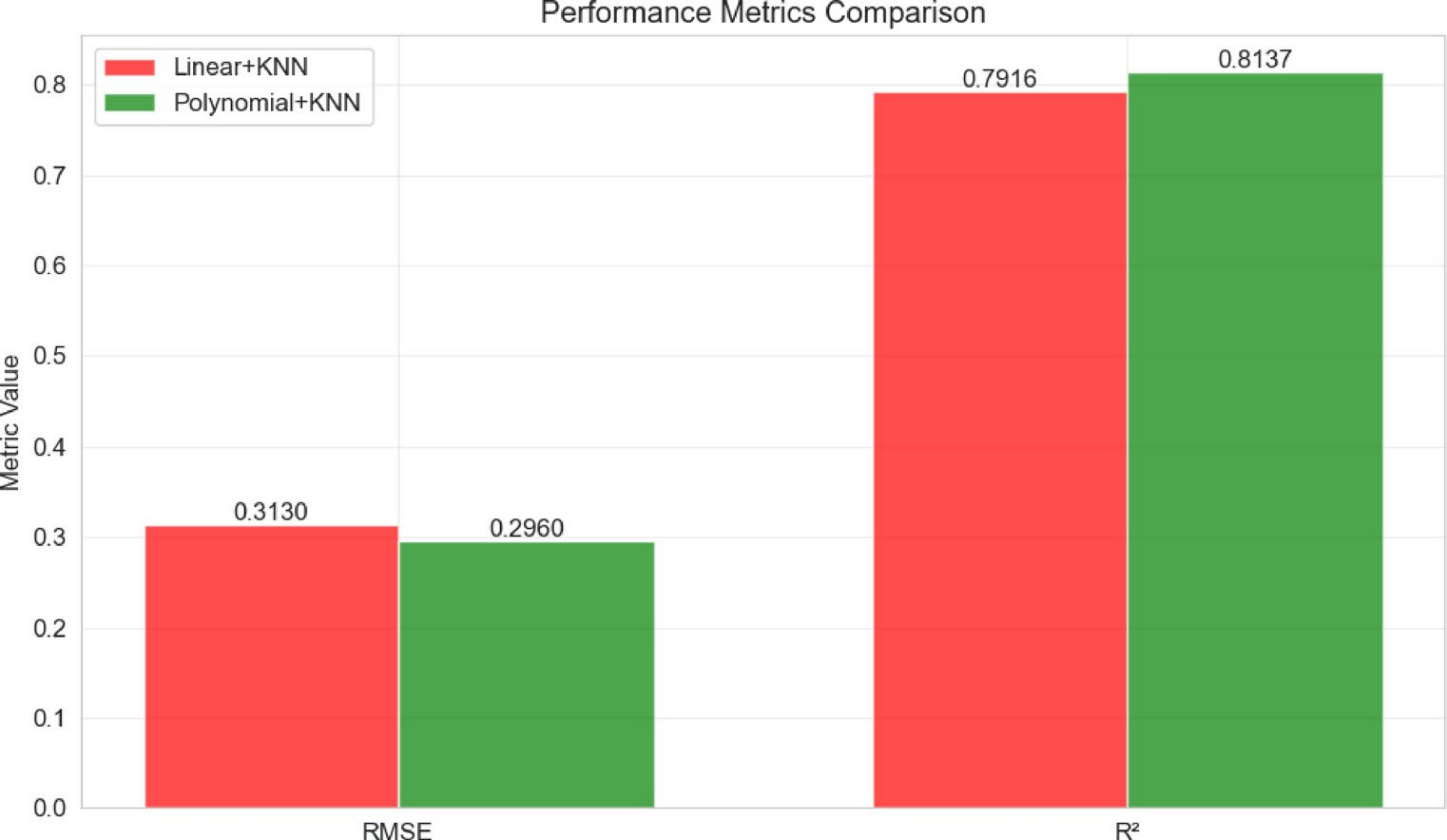
Performance comparison of hybrid ML models for real-time hardware bias prediction.

A comparative analysis of the two proposed hybrid ML models intended for real-time hardware bias prediction in optical wireless communication systems is shown in Fig. 11. Performance metrics, RMSE and R^2^, are visualized in a grouped bar chart, making it easy to directly compare the Hybrid Linear Regression with KNN and Hybrid Polynomial Regression with KNN models. The results show that both hybrid architectures achieve robust predictive performance, with Hybrid Linear with KNN and Hybrid Polynomial with KNN modes yielding RMSE values of 0.3130 and 0.2960, respectively, which corresponds to a 5.4% reduction in prediction error for the polynomial-based approach. Correspondingly, the R^2^ scores of 0.7916 and 0.8137 indicate that both models explain a substantial proportion of variance in hardware bias, with the hybrid Polynomial with KNN model capturing approximately 2.8% more variance than its linear counterpart.

**Fig. 12:**
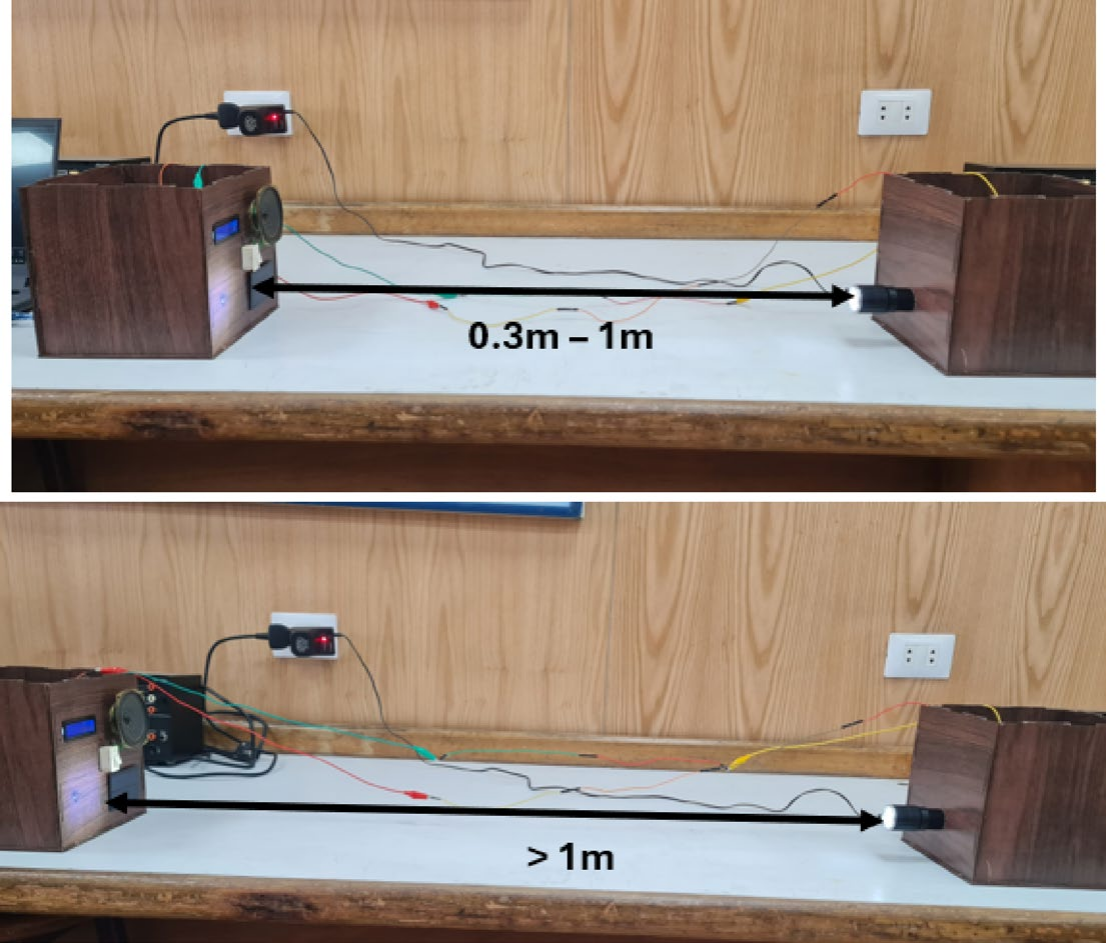
Experimental setup illustrating the proposed system, tested at different transmission distances.

To verify the feasibility of the proposed system over extended transmission distances, additional experiments were conducted using the same experimental setup while varying the transmitter–receiver separation. Figure 12 presents the experimental arrangement at different link distances, confirming that the system was physically tested beyond the nominal operating range considered in the main performance analysis.

**Fig. 13:**
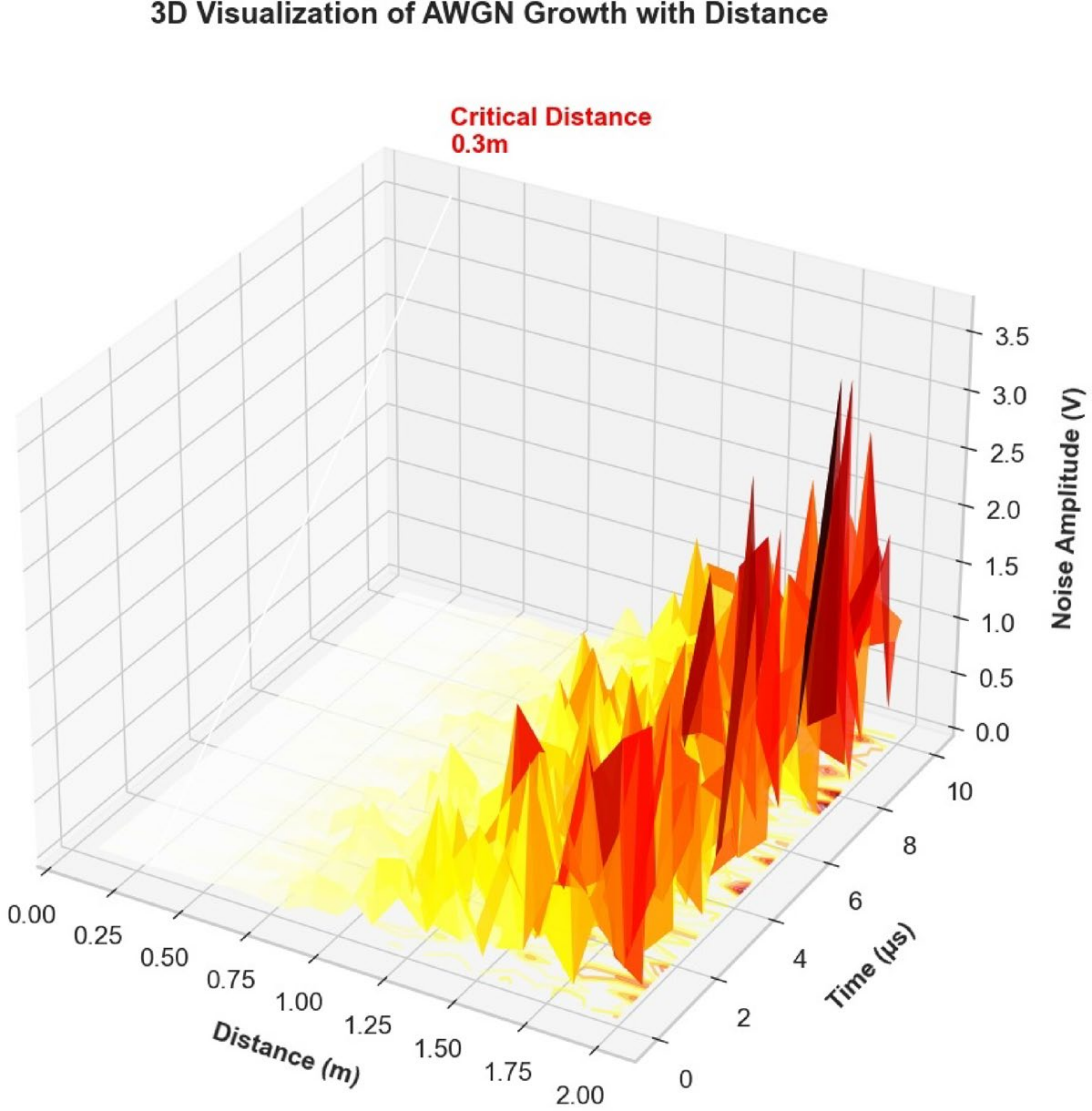
Three-dimensional characterization of AWGN propagation in hardware design.

As AWGN in optical wireless communication systems increases with transmission distance, Fig. 13 provides a three-dimensional visualization of the family of noise observed. The Heatmap represents: the vertical axis is noise amplitude (V), the horizontal axes are transmission distance (m) and temporal variation (µs); color intensity means noise magnitude. A clearly visible threshold plane, which is 0.3 m long, determines whether operations on board or ashore will be affected by noise before and after its demarcation line is crossed. Based on the noise characterization presented:

- Short-Range Optimization (< = 0.3 m): Systems can be optimized for maximum spectral efficiency with minimal error protection overhead. Receiver designs can prioritize linearity over sensitivity.
- Medium-Range Adaptation (0.3–1.0 m): Implementation of adaptive power control and modulation schemes becomes essential.
- Long-Range Operation (> 1.0 m): Requires sophisticated noise mitigation including advanced equalization techniques, diversity combining (spatial, temporal, or frequency), Machine learning-based channel estimation, adaptive filtering with noise prediction.

The 0.3-m threshold emerges as a key design parameter for practical Li-Fi system deployment, balancing performance objectives with implementation complexity.

## Conclusions

In conclusion, this paper successfully identified the optimal DC bias value for DCO-OFDM-based Li-Fi systems using machine learning algorithms. By comparing different ML techniques, such as hybrid linear regression with KNN, hybrid polynomial regression with KNN, ridge regression, hybrid linear and ridge regression, hybrid polynomial and ridge regression, and Bayesian regression, we observe that hybrid polynomial regression with KNN outperforms the other models, with a lower RMSE value of 0.18847, a higher R2 score of 96.908, and a lower MAPE of 9.271% on the MATLAB dataset. Furthermore, hardware experiments were conducted to validate the ML models using real-time data. The results confirmed that hybrid polynomial regression with KNN maintained its superior performance on both simulated and hardware-collected data.

These findings suggest that the hybrid polynomial regression with KNN is an effective and reliable method for optimizing DC bias in DCO-OFDM-based Li-Fi systems, thereby minimizing inefficiencies like clipping noise and reduced optical power.

This work has a few limitations. The hardware validation was performed at a distance of only 0.3 m, and long ranges (above 0.3 m) aren’t considered yet due to resource limitations, which may be upgraded in future work and may not fully reflect practical indoor Li-Fi environments. The dataset combined simulation and limited measurements, without considering effects such as multipath or ambient light interference.

Future work will extend this work beyond DCO-OFDM to include alternative modulation schemes such as ACO-OFDM and Flip-OFDM, enabling broader validation of the proposed models based on Software defined radio such as USRP 2920^37^. In addition, experiments can be conducted over longer transmission distances and with enriched features, such as the channel impulse response and ambient light variations, to better capture practical Li-Fi conditions.

## Data Availability

Our code is open-source and available at the following GitHub repository: https://github.com/ahh001/dc-bias-optimization-lifi-and-validation.git. Zendo: 10.5281/zenodo.19593816
